# Quality Assessment of Goldenrod, Milkweed and Multifloral Honeys Based on Botanical Origin, Antioxidant Capacity and Mineral Content

**DOI:** 10.3390/ijms23020769

**Published:** 2022-01-11

**Authors:** Marianna Kocsis, Alexandra Bodó, Tamás Kőszegi, Rita Csepregi, Rita Filep, Gyula Hoffmann, Ágnes Farkas

**Affiliations:** 1 Institute of Biology, Faculty of Sciences, University of Pécs, 7624 Pecs, Hungary; alexandrabodo88@gmail.com (A.B.); hgyula@gamma.ttk.pte.hu (G.H.); 2 Department of Laboratory Medicine, Medical School, University of Pécs, 7624 Pecs, Hungary; koszegi.tamas@pte.hu (T.K.); ritacsepregi93@gmail.com (R.C.); 3 János Szentágothai Research Center, University of Pécs, 7624 Pecs, Hungary; 4 Department of Pharmacognosy, Faculty of Pharmacy, University of Pécs, 7624 Pecs, Hungary; rita.filep@gmail.com (R.F.); agnes.farkas@aok.pte.hu (Á.F.)

**Keywords:** honey, pollen spectrum, antioxidant capacity, multielement content, PCA analysis

## Abstract

The goal of the study was to evaluate the pollen spectrum, antioxidant capacity and mineral content of four Hungarian honey types, using multivariate statistical analysis. The light colored honeys were represented by milkweed honey and a multifloral (MF) honey with dominant pollen frequency of linden (MF-*Tilia*); the darker ones were goldenrod honey and a multifloral honey with Lamiaceae pollen majority (MF-Lamiaceae). The pollen spectrum of the samples was established with melissopalynological analysis. The absorbance of the honeys positively correlated with the antioxidant capacity determined with three of the used methods (TRC, TEAC, DPPH), but not with ORAC. The latter method correlated negatively also with other antioxidant methods and with most of the mineral values. MF-*Tilia* had high ORAC value, K and Na content. The MF-Lamiaceae had the highest K, Mg, P, S, Cu and Zn content, the last five elements showing strict correlation with the TRC method. The darker goldenrod honey had higher SET values and total mineral content, than the milkweed honey. The above character-sets facilitate identification of each honey type and serve as indicators of variety. The antioxidant levels and mineral content of honeys allowed their clear separation by principal component analysis (PCA).

## 1. Introduction

Honey is a natural product with significant nutritional and medicinal benefits due to its strong antibacterial and antioxidant activity. The antioxidant substances of honey, such as polyphenol compounds, vitamins and multielements are transferred from plants by bees [1]. These plant antioxidants are highly bioactive secondary metabolites, including the most abundant group, the phenolics, with the highest antiradical activity [2].

It is well known that main quality parameters of honey depend primarily on the botanical origin. Several attempts have been made to develop rapid methods for evaluation of the floral origin of honey, since the principal method, the melissopalynological analysis, requires time and considerable botanical knowledge [3]. However, extensive palynological studies of different honey types do exist, which determined the botanical origin of several uni- and multifloral honeys in different countries [4,5,6]. In case of multifloral honeys, pollen analysis is still inevitable to establish their diverse botanical origin, which in turn can be related to other parameters of the honey, such as physicochemical characters or antioxidant activity.

The relationship between floral origin and other properties of honey, like antioxidant or antibacterial activities, is well established [7,8]. Dżugan et al. (2018) [9] found that antioxidant capacity can be a useful indicator for the botanical origin of honey. There are several analytical methods for determination of its bioactivity. All of them have advantages and disadvantages and only the combination of these methods can provide accurate results. The assays for determining antioxidant capacity can be divided into single electron transfer (SET) and hydrogen atom transfer (HAT) methods [10]. The most commonly used assays include SET methods, such as Total Reducing Capacity (TRC) measured by Folin-reagent, Trolox Equivalent Antioxidant Capacity (TEAC), 1,1-diphenyl-2-picrylhydrazyl (DPPH) assay and the Ferric Reducing Antioxidant Power (FRAP). In contrast, the oxygen radical absorbance capacity (ORAC) assay based on HAT method has been used less frequently with honeys. Maurya et al. (2014) [11] and more recently Martinello et al. (2021) [12] summarized a remarkable amount of antioxidant parameters of different honey types in their comprehensive reviews, among others several multifloral honeys, from different floral and geographical origin. 

Macro- and microelements also play an important role in the quality of honey. Its mineral composition partly depends on the soil and has strong botanical specificity. In Solayman et al.’s (2016) [13] review the mineral content reported in honeys has been compared among countries. They have found that the mineral composition of honey varied depending on botanical and geographical origins throughout the world. Recent studies were directed at the analysis of mineral content in honeys focusing on Hungarian [14], Croatian [15] and Italian multifloral honeys [16]. Mohammed et al. (2018) [17] assigned the element content of Yemeni honeys as a long-lasting marker to ascertain honey floral origin and quality.

Many studies reported that antioxidant capacity of honeys strongly correlated with its color, and there was also strong correspondence between the results of different antioxidant assays [8,9,18,19]. The correlation between honey color and mineral content has also been observed [13]. Fewer studies were devoted to analyzing the correlation between antioxidant activity and minerals [20,21,22]. Principal component analysis (PCA) was successfully used for characterization of honeys based on several factors, such as mineral, physicochemical and enzymatic analysis; antioxidants and physicochemical properties; metal content and contamination (antibiotic and pesticides residues); browning index and antioxidant activity [21,23,24,25], respectively. 

Based on the observation that the color of honey can be an indicator of antioxidant capacity and mineral content, two light colored and two medium dark uni- and multifloral Hungarian honey types were selected for the purposes of the present study. The botanical sources of unifloral honeys were milkweed (*Asclepias syriaca*) and goldenrod (*Solidago gigantea*), both plants being native to North America, and invasive species in Hungary. The aim of our study was to differentiate these four honey types based on their sensory characteristics, pollen spectrum, color, macro- and microelement content, and antioxidant capacity determined with four different assays. A further objective was to reveal if there are correlations between the above characters. As a third goal, PCA was conducted to determine the discriminating power of absorbance, antioxidant values, macro- and micromineral content. 

## 2. Results

### 2.1. Sensory Characteristics, Color and Pollen Analysis of Honey Samples

Honey samples were evaluated and identified based on sensory characteristics, such as odor, consistency and spectrophotometric color determination (Table 1). Furthermore, detailed melissopalynological analysis was carried out to reveal the pollen spectrum of the samples and to determine their floral origin (Table 2). 

The milkweed honey was classified as unifloral honey based on its sensory characteristics. It has a special aromatic, pleasant scent that is kept by the honey for months. Its color was reported very similar to the pale, yellowish green color of robinia honey [26], while in a comparative study of milkweed and robinia honeys, milkweed honey proved to be of darker color [27]. Accordingly, in our study, the color of milkweed honey was darker and located between the light beige phacelia and the light amber linden honeys [22]. In case of this special honey, the pollen analysis cannot prove its botanical origin, because honeybees do not harvest pollen grains of this plant. Although the analysis revealed rape pollen as dominant pollen type, with relative frequency slightly above 45%, this honey should not be considered a unifloral rape honey, in which rape pollen has to be overrepresented, requiring a minimum of 60% of *Brassica* sp. pollen grains [15], or even above 80%, based on our previous study [28]. 

The sensory characteristics and pollen spectrum of goldenrod honey with *Solidago* as the dominant pollen type, proved the unifloral origin of this honey type. It had rich amber color, much darker than reported by Kuś et al. (2014) [29] and Jasicka-Misiak et al. (2018) [19]. They described the Polish unifloral goldenrod honey as extra light amber, semisolid, fine granulated, with the color parameter of 138–205 mAU. The explanation for the significant color difference could lie in the botanical source of Polish goldenrod honey, which is the native European goldenrod (*Solidago virgaurea*) species. Piljac-Žegarac et al. (2009) [30] measured 287 mAU of Croatian goldenrod honey with the same botanical origin. In our study, the darker colored honey (535 mAU) originated from the North American invasive species (*Solidago gigantea*). Our observation was supported also by the description of goldenrod honey from the U.S. (Illinois), claiming that the color of goldenrod-based honey is a rich amber, almost as dark as a maple syrup [31].

The light and dark colored multifloral honeys in our study showed remarkable differences in their pollen spectrum, as expected. The most abundant pollen type of the lighter colored multifloral honey was *Tilia*, while the darker colored one was dominated by Lamiaceae pollen (Table 2). Multifloral honeys obviously represent a multicolored repertoire with countless pollen composition, providing diverse features from all aspects, regarding among others antioxidant parameters or mineral content [11,12,16].

### 2.2. Total Antioxidant Capacities of Honeys

Combination of non-enzymatic antioxidant assays provides the most reliable results. Therefore, we used four different tests to determine the bioactivity of the honeys studied (Table 3). The results of the SET based methods—TRC, TEAC and DPPH—showed parallel tendency with the darkness of honeys. TRC distinguished milkweed, goldenrod and MF-Lamiaceae, but MF-*Tilia* was not significantly different from the unifloral honeys. Light and dark colored samples were clearly separated by the TEAC results. DPPH assay had the lowest distinctive power among the honeys studied. The HAT based method, ORAC, separated the uni- and multifloral honeys from each other. The highest SET based antioxidant capacities were measured in the dark colored MF-Lamiaceae honey, while the lowest values were determined for milkweed honey. Regarding ORAC results, MF-*Tilia* had the highest and goldenrod honey the lowest antioxidant activity. 

Several researchers have chosen multifloral honeys in their study without analyzing their pollen spectrum or determining their absorbance. Nevertheless, in our complex analysis we made an effort to compare them based on their antioxidant parameters as well. In Maurya et al.’s (2014) [11] overview on antioxidative capacity of honeys from different countries, the range of TRC of multifloral honeys was between 32 and 147 mg GAE kg^−1^. Sreckovic et al. (2019) [8] measured lower TRC of a multifloral honey (87 mg GAE kg^−1^) compared to our multifloral ones. The Polish multifloral honeys involved in Sawiczki et al.’s (2020) [32] and in Dżugan et al.’s study (2018) [9] had similarly high TRC values as our MF-Lamiaceae honey. In our study, the MF-*Tilia* and MF-Lamiaceae presented similar TRC values as the Turkish yellowstar-thistle and parsley honeys, respectively [18]. Predominantly higher TRC values (325–937 mg GAE kg^−1^) were measured in 18 multifloral honeys from Burkina Faso (Africa) by Meda et al. (2005) [33] compared to the result of MF-Lamiaceae provided by our study. Comparing the TRC results of our goldenrod honey with the Polish goldenrod honey from a different *Solidago* species, the latter gave significantly lower values (147–199 mg GAE kg^−1^) [29], but similar values were measured by Jasicka-Misiak et al. (2018) [19] (210.3 mg GAE kg^−1^), and higher values of Croatian goldenrod honey (*Solidago virgaurea* L.) by Piljac-Žegarac et al. (2009) [30] (492 mg GAE kg^−1^). In Dżugan et al.’s (2018) [9] study, goldenrod honey was an exception in the color-TRC correlation, because despite its light color, its antioxidant activity was comparable to that of the dark honeydew honey.

The TEAC values of our honeys were lower than those of the Serbian dark colored unifloral honeys, such as meadow honey (352 μmol TE 100 g^−1^) or forest honey (585 μmol TE 100 g^−1^), but higher than the light colored acacia (102 μmol TE 100 g^−1^) [34]. Our honey samples were in the middle of the radical scavenging ability range of the Sicilian black honeybee unifloral honey samples, starting from tangerine (19.2 μmol TE 100 g^−1^) to dill honey (270.3 μmol TE 100 g^−1^) [35]. The values of our honeys were higher than those of the Brazilian stingless bee unifloral honeys [36]. The antioxidant capacity of milkweed honey was similar to the early spring multifloral honey measured in our previous study [28]. 

The DPPH range was more limited than TRC or TEAC. The explanation for the higher activity range observed with TEAC (ABTS) than with DPPH was that TEAC (ABTS) radical reacts with both hydrophilic and lipophilic antioxidants, while DPPH only with lipophilic ones [19]. The IC_50_ values of the DPPH analysis were lower for the dark multifloral honey in this study, which means that its antiradical power was significantly higher than that of the other honeys. The DPPH values of our honeys were around the range of the DPPH values of linden and sunflower honeys [22]. Slovenian and Turkish linden (*Tilia*) honeys showed similar DPPH value as our MF-Lamiaceae honey, while the others with lower antioxidant activity showed similar values to the Turkish yellowstar-thistle honey [18,37]. Beretta et al. (2005) [38] reported lower antioxidant values in unifloral dandelion and acacia honeys, while their multifloral honey was much more active (IC_50_ = 5.32 ± 0.2 mg mL^−1^) compared to our honeys in this study.

ORAC is thought to be the most biologically relevant assay, which is based on hydrogen atom transfer [12], thus it may evaluate different groups of antioxidants than the previous three. Goldenrod honey provided similarly low ORAC value as acacia honey, while the MF-*Tilia* and MF-Lamiceae honeys presented significantly higher antioxidant activity, as the dark colored fennel and the amber colored sunflower, respectively [22]. Our milkweed honey showed a similar ORAC value as that of the strawberry tree honey, furthermore the values of our other honeys were significantly higher even than that of the dark African or buckwheat honeys [38]. Serbian unifloral honeys also showed lower ORAC activity compared to honeys in this study, while all of the Brazilian stingless bee honeys had higher values [34,39].

### 2.3. Multielement Analysis of Honeys

**Figure 1:**
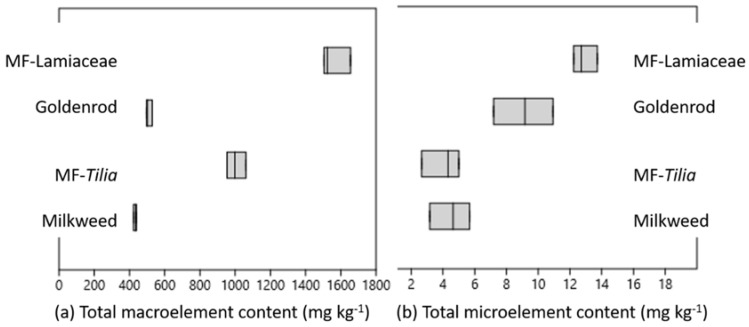
Total (**a**) macro- (K, Ca, P, S, Mg, Na) and (**b**) microelement (B, Cu, Fe, Mn, Zn) content of the honeys studied.

The macro- and microelement contents determined in the honey samples are summarized in Table 4 and Table 5 and Figure 1. Detailed mineral parameters of our samples from the southern part of Hungary (Southern Transdanubia) were compared primarily with the mineral content of honey samples mainly from the eastern part of the country (Hungarian Great Plain) [14,40,41]. The most abundant macroelement was K in the honeys, expectedly, but the other macroelements followed different decreasing quantity order in different honeys. Ca was the second most abundant macroelement, except in milkweed honey. In milkweed and MF-Lamiaceae S content was higher than Mg, MF-*Tilia* contained a similar amount of S and Mg, and goldenrod had more Mg content than S. The total macroelement content significantly separated the unifloral honeys from the MF honeys, and the MF honeys from each other (Figure 1a).

There were also significant differences between the K content of uni- and multifloral honeys, and even between that of the multifloral honeys. MF-Tilia and MF-Lamiaceae honeys had similar K content as sunflower and linden honeys, respectively [14]. Significantly lower K content characterized the milkweed and goldenrod honeys, similarly to acacia, phacelia and rape honeys originated from the Eastern part of the country. The Ca content in case of goldenrod and MF-Lamiaceae, and the P, S, Mg content in MF-Lamiaceae was significantly higher than that in the honey types analyzed by Czipa et al. (2015) and Sajtos et al. (2019) [14,41], except sunflower honey. The Na content of MF-Tilia was surprisingly high compared to the honey types in the present and in our previous studies [22]. With regard to non-Hungarian honeys, the range of their Na content differed significantly between continents, e.g., honeys from Europe (Bulgaria, Italy, Poland, Spain) contained a relatively low amount of Na (7.2–152 mg kg^−1^), compared to the Na levels of honeys detected in India or Malaysia (83–732 mg kg^−1^) [13]. The Na level depends also on honey type, e.g., exceptionally high Na content (279 mg kg^−1^) characterized the avocado honey in Spain [42].

All of the honeys contained B, while Fe, Mn and Zn were under the detection limit in some of the light honey samples (Table 5). The dark-colored honeys had significantly higher total microelement content than the light honeys (Figure 1b), but there were differences in the ranking of each element. MF-Lamiaceae was the richest in B, Cu, Mn and Zn, while high Fe content characterized the goldenrod and MF-Lamiaceae honeys. The multifloral honeys were distinguishable from the unifloral ones due to their significantly higher Mn content, while Fe and Zn content significantly separated the light and dark colored honeys from each other. 

The B content in goldenrod and MF-Lamiacae was similar to sunflower and fennel honeys in our previous study [22]. Comparing the content of other trace elements in our honeys to that of honeys studied by Hungarian researchers provided the following observations [14,22,41]: low Cu content was measured in milkweed, MF-*Tilia* and goldenrod honeys in this study, similarly to that of the light-colored honeys like acacia, amorpha and phacelia honeys, while MF-Lamiaceae showed about 6-times higher Cu content. Fe content of milkweed and MF-*Tilia* was similar to multifloral and sunflower honeys, respectively. Higher Fe content was found in MF-Lamiaceae, and similar amount was measured in a rape honey. Our unifloral honeys gave low Mn content similarly to the light colored unifloral acacia, amorpha and phacelia honeys, while that of our multifloral honeys was significantly higher, close to the values of rape and sunflower honeys. Zn content showed a similar trend to what we observed in case of light and dark colored unifloral honeys from Transdanubian region of the country, while the light acacia and rape honeys from the eastern part of Hungary had significantly higher Zn content, similar to our darker goldenrod and MF-Lamiceae honeys. The results of multielement analysis in Egyptian and Italian honeys proved that mineral content can serve also as a marker of geographical origin [16,43,44]. 

### 2.4. Multivariate Analysis

**Figure 2:**
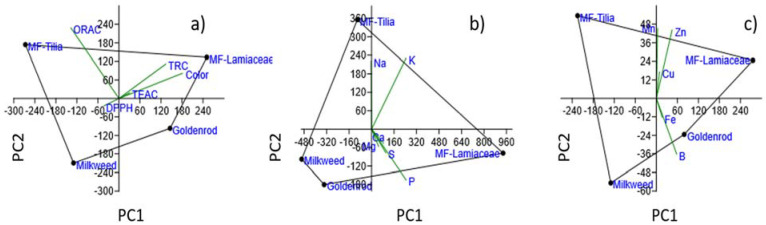
Principal component analysis (PCA) of dataset consisting of (**a**) antioxidant and color parameters, (**b**) macroelements and (**c**) microelements analyzed from the honey samples.

The correlation matrix with *p*-tests of color, antioxidant values and multielement contents was analyzed by Pearson’s correlation in order to enlighten the possible link between the studied parameters. PCA revealed the complex indicator role of parameter groups in identification and differentiation of honey types (Table 6, Figure 2). 

The SET based antioxidant methods highly correlated with each other and with the color of honey, consistently with the results of other studies [8,19,29,45]. The TEAC assay was the strongest predicting factor regarding the color of the honey samples, supported also by Dżugan et al. (2018), Flanjak et al. (2016) [9,46]. Many studies described that darker honeys had higher antioxidant values, while lighter honeys were characterized by relatively low ones [38]. However, ORAC as an exception, showed positive correlation only with DPPH, in contrast to the results reported by Beretta et al. (2005), Gorjanovich et al. (2013), Bodó et al. (2021) [22,34,38]. Halagarda et al. (2020) [47] also reported a positive dependence between ORAC and TRC (indicated as total polyphenolic content (TPC)). However, Küçük et al. (2007) [48] noted the inconsistency between the polyphenolic content and the antioxidant activity. It seems that our sample selection revealed the important additional role of the HAT method besides the SET assays. 

Regarding the macro- and microelement content, color gave a high positive correlation with the amount of several minerals, which further supported their contribution to determine honey color [49]. TRC had strict correlation (r ˃ 0.9) with P, S, Mg and Cu further proving their possible link [22]. Mg, B and Zn highly correlated with the SET antioxidant assays, Fe with the color and TEAC assay. ORAC had correlation with K, Na and Mn. 

Three groups of parameters—color with antioxidant assays, macroelements, microelements—were selected for PCA in order to establish their identification power in terms of honey types. The results are presented in the biplots of Figure 2.

The first principal component, PC1, included most of the information with 93.75% for the antioxidant results, 99.25% for the macroelements and 92.52% for the microelements of the total variance, while the second principal component, PC2, explained 5.69%, 0.48%, 4.34%, respectively. The dark colored honeys with higher SET based activities were located on the positive PC1, while light colored honeys with lower parameters were characterized by negative PC1 values of the plot. The multifloral honeys had positive PC2 values, while uniflorals were on the negative PC2 coordinate. In this parameter group the ORAC activity was useful in clustering uni- and multifloral honeys separately. In case of macroelements, MF-Lamiaceae represented positive PC1 value, and MF-*Tilia* positive PC2 value. Na and K played a key role in the clustering of MF-*Tilia*, P and S in that of MF-Lamiaceae. The third group, the microelements, separated clearly the honey types. Light and dark colored honeys were differentiated by B and Fe content, while uni- and multifloral honeys by Cu, Mn and Zn content. 

Several attempts have been made to identify and differentiate honey types from each other by means of PCA. Nayik et al. (2018) [21] also revealed that PCA of antioxidant properties and minerals successfully clustered three Indian honey types, while their sugar content did not provide as so information. Besides the antioxidant activities and mineral contents, values of vitamin B2 classified Turkish honeys from different botanical origin [20]. Scripcă et al. (2021) [24] also performed chemometric methods to emphasize the differences among eight Romanian honey types, based on mineral content, color, antibiotic and pesticide residues. However, the PCA of physicochemical parameters was only partly successful in identification of honey types with similar characteristics from different regions in Romania [23]. The light and dark colored honeys from Poland could be successfully differentiated using PCA with nine variables (browning index, color parameters and antioxidant values) [25].

## 3. Materials and Methods

### 3.1. Samples

The honey samples were purchased from local producers in Hungary in 2018; the multifloral and goldenrod honeys were harvested in the Southwest Transdanubium area, milkweed honey originated from the Southern Great Plain area. They were stored at room temperature (20–21 °C) in the dark for a maximum of three weeks. For each honey type (Table 1), measurements were carried out on 3 parallel samples; altogether, 12 honey samples were analyzed.

### 3.2. Melissopalynological Analysis

Frequency determination of the most important pollen types was executed according to the modified Von Der Ohe et al.’s (2004) [50] method. Ten grams of each honey included in analysis was homogenized and dissolved in 20 mL warm distilled water (not above 40 °C), then centrifuged for 10 min (3000 r min^−1^) with a Neofuge 15R centrifuge (Lab-Ex Ltd., Budapest, Hungary) and the supernatant liquid was decanted, then the sediment was dispersed again with 10 mL distilled water, and centrifuged for 5 min (3000 r min^−1^). A 20 × 20 mm square was marked on a slide, and the sediment was smeared in this area. Two slides were prepared from each sample. The slides were dried at 40 °C on a heating plate (OTS 40, Tiba Ltd., Győr, Hungary), then covered with a few drops of fuchsine glycerol jelly (fuchsine added to Kaiser’s glycerol gelatine, Merck Life Science Ltd., Budapest, Hungary). For quantification of pollen types, at least 400 pollen grains were counted from the samples under a light microscope (Motic BA310, Electromed Kft., Budapest, Hungary) at 400× magnification. The frequency of the pollen types was calculated in all the honey samples in percentages. 

### 3.3. Determination of Color Intensity (ABS_450_)

Color intensity was measured according to Beretta et al.’s (2005) [38] protocol. Honey was diluted to 50% (*w*/*v*) with warm water (45–50 °C), sonicated for 5 min, filtered (0.45 µm pore size, Agilent Technologies, Milan, Italy) and the net absorbance was defined as the difference between spectrophotometric absorbance at 450 and 720 nm. The absorbance was measured using a Shimadzu UV-1800 spectrophotometer (Shimadzu Schweiz GmbH, Reinach, Switzerland), and the results were expressed as a milli-absorbance unit (mAU). 

### 3.4. Determination of Total Reducing Capacity (TRC) 

TRC was determined by the Folin–Ciocalteau method as reported by Singleton et al. (1999) [51] with minor modifications. Honey samples (0.1 g) were diluted to 1 mL with distilled water and 0.5 mL of the solution was added to 100 µL of 10% Folin–Ciocalteu reagent, 300 µL distilled water and 400 µL 6% Na_2_CO_3_ solution. The absorbance was determined after 20 min at 760 nm. The results were expressed as mg of gallic acid per kg of honey (mg GAE kg^−1^). A total of 50–200 µg mL^−1^ gallic acid was used as a standard to establish the calibration curve. The chemicals were obtained from Merck Life Science Ltd., Budapest, Hungary. 

### 3.5. Determination of Trolox Equivalent Antioxidant Capacity (TEAC)

The TEAC assay was carried out using the method of Re et al. (1999) [52]. Honey was diluted in distilled water with a ratio of 1:10. 2,2″-Azino-bis(3-ethylbenzothiazoline-6-sulfonic cation radical) (ABTS^+^) was prepared by mixing 0.1 mM ABTS, 0.0125 mM horseradish peroxidase and 1 mM H_2_O_2_ in a 50 mM phosphate buffer pH 6.0. After 15 min, 100 μL diluted honey was added to 1 mL ABTS^+^ solution and the reduction of absorbance at 725 nm was measured. For calibration Trolox (0.004–0.02 μM) was used. The results were expressed as μmol TE 100 g^−1^ honey. The chemicals were obtained from Merck KGaA (Darmstadt, Germany). 

### 3.6. Measuring the Antiradical Power (DPPH)

The antiradical activity of samples was measured using the method of Beretta et al. (2005) and Bertoncelj et al. (2007) [37,38] with minor modifications. In total, 4 mg of DPPH (Merck Life Science Ltd., Budapest, Hungary) in 50 mL of 96% absolute ethanol (200 µmol L^−1^, Reanal Labor, Budapest, Hungary) was prepared and kept at 5 °C. Trolox (Merck Life Science Ltd., Budapest, Hungary) standards were prepared in 100 mM acetate buffer at pH 5.5 (100 mM acetic acid and 100 mM sodium acetate trihydrate, Reanal Labor, Budapest, Hungary), in the concentration range of 0–180 µmol L^−1^. The assay was adapted to a plate reader (Perkin Elmer EnSpire Multimode reader, Waltham, MA, USA) using standard 96-well plates (Sarstedt AG & Co. KG, Nümbrecht, Germany). Into each well, 50 μL of the blank/standard/sample, 95 μL of DPPH solution and 50 μL of acetate buffer solution were added. The mixture was shaken and the absorbance changes were measured at 517 nm after 60 min of incubation in the dark at room temperature (25 °C). The radical-scavenging activity was expressed as IC_50_ (the concentration of the honey sample (mg mL^−1^) needed to scavenge 50% of DPPH), calculated by using a linear regression analysis.

### 3.7. Oxygen Radical Absorbance Capacity (ORAC)

The ORAC assay was based on the procedure previously described by Kőszegi et al. (2017) and Patay et al. (2016) [53,54] without modifications. In summary, a fluorescein working solution (400 nmol L^−1^, Merck Life Science Ltd., Budapest, Hungary) and the 2,2′-azobis(2-amidinopropane) dihydrochloride (AAPH) oxidant (400 mmol L^−1^, Merck Life Science Ltd., Budapest, Hungary) dissolved in 75 mmol L^−1^ potassium phosphate buffer (mixture of KH_2_PO_4_ and K_2_HPO_4,_ Reanal Labor, Budapest, Hungary) at pH 7.5 were prepared freshly before the measurements. Trolox standards were prepared in the potassium phosphate buffer (0–160 µmol L^−1^). Into each well, 25 µL of the blank/standard/sample and 150 µL of fluorescein solution were added in optical plates (Perkin Elmer) and the mixture was incubated at 37 °C for 30 min in the dark. Next, 25 µL AAPH solution/well was injected by the automated injector of a Biotek Synergy HT plate reader (BioTek Instruments, Winooski, VT, USA) previously warmed up to 37 °C. The fluorescence intensities were monitored for 80 min (490/520 nm wavelengths) at 2 min intervals. The area under each curve (AUC) was obtained using the software of the reader providing the total sum of the individual digital data of the corresponding fluorescence signals. The antioxidant capacity values were expressed as µmol Trolox equivalent (TE) g^−1^ honey.

### 3.8. Inductively Coupled Plasma Atomic Emission Spectrometry (ICP-AES)

ICP-AES measurements of 20 elements were completed using an ICPE-9000 instrument (Shimadzu, Kyoto, Japan) at the following operating parameters: radio frequency power, 1.20 kW; plasma gas, 10.0 L min^−1^; auxiliary gas, 0.60 L min^−1^; carrier gas, 0.70 L min^−1^; and view direction, axial. Prior to the elemental analysis, honey samples were pretreated using a Multiwave 3000 (Anton Paar GmbH, Graz, Austria) microwave system, in which 1 g of each honey sample was treated in three steps: 300 W for 5 min, 1000 W for 5 min and 1400 W for 20 min. The instrument was calibrated using inorganic reference standards for a single element (BDH Prolabo Chemicals, VWR International Kft., Debrecen, Hungary). Quality control was assured using a high-purity multielement standard solution containing 25 elements (HPS, RK Tech Kft., Budapest, Hungary). A recovery test was undertaken by spiking rape honey with 20 ppm of the ICP multielement standard mixture. Recoveries for the 20 elements ranged from 93.8% to 111.5%. All analyses were carried out in triplicate. Detection limits (LOD) were as follows: 15.0 mg kg^−1^ for K, 10.0 mg kg^−1^ for Ca, 5.0 mg kg^−1^ for S and Na, 2.0 mg kg^−1^ for Mg, 1.5 mg kg^−1^ for P, 1.0 mg kg^−1^ for B and Al, 0.5 mg kg^−1^ for Fe and Pb and 0.1 mg kg^−1^ for As, Cd, Co, Cr, Cu, Mn, Mo, Ni, V and Zn.

### 3.9. Statistical Analysis

All measurements were completed on three biological replicates of four honey types. Statistical analyses were carried out using Excel^®^ (Microsoft Corp., Redmond, WA, USA) and the PAST software package version 3.11 [55] at a 5% or 1% significance level (*p* < 0.05, *p* < 0.01), after normality checking with the Shapiro–Wilk test. For the correlation matrix, moreover, the 0.1% (*p* < 0.001) significance level was used to indicate the greater significance of the differences. Data were expressed as means ± standard deviations (SD). Pairwise comparisons were performed with Student’s t-tests. Interactions between the measured parameters were investigated with Pearson’s rank correlation using PAST. To describe relatedness among honey types, we performed a centered and standardized principal component analysis (PCA) with all measured parameters. Distances among object points (honey types) were calculated with Euclidean distances.

## 4. Conclusions

The honey types selected for this study were successfully distinguished based on the studied parameters. The correlation matrix interpreted the relationships between absorbance, antioxidant values, macro- and micromineral content, while PCA was used to illustrate the discriminating power of three groups of characters. The honey types were placed on different PC plots by antioxidant results completed with the color and microelement content. There is clear evidence for the importance of the HAT based method besides the SET assays to reveal unique features of the honey type. The mineral content gave also useful information on the honeys studied, however, it may depend not only on the botanical, but also on the geographical origin. Color as an easily detectable and important feature of honey, can refer to its bioactivity and mineral content as well, but their detailed analysis provides special characters of the given honey type. In case of multifloral honeys the melissopalynological analysis is inevitable, while unifloral honeys can be usually well identified by physicochemical characters. Although unifloral honeys represent a bigger value on the market than multifloral ones, there is a great potential in the latter, due to their multivariate botanical origin, which may give rise to unique quality properties. 

We can conclude that the revealed character sets provide a useful identification tool to the given honey type, reflecting its floral origin and quality. Furthermore, there are possible relations among the studied parameters suggested by this multi-level study.

## Figures and Tables

**Table 1:** Identification, color and sensory characteristics of analyzed honey samples.

Nr.	Honey Type Plant Name	Sensory Characteristics (Color, Odor and Consistency)	ABS_450_ (mAU)
1	Milkweed *Asclepias syriaca*	Light yellowish amber, moderately intense flower-like odor, liquid, viscous	245 ± 12
2	Multifloral-*Tilia* (MF-*Tilia*)	Light amber, intense odor, semisolid, fine granulated	306 ± 8
3	Goldenrod *Solidago gigantea*	Amber, moderately intense odor, semisolid, fine granulated	531 ± 15
4	Multifloral-Lamiaceae (MF—Lamiaceae)	Brownish amber, intense malt odor, semisolid, fine granulated	606 ± 18

Each code number in the first column represents three biological replicates (*n* = 3) of honey types.

**Table 2:** Pollen spectrum of the studied honeys.

Pollen Type Relative Frequency (%)	Milkweed Honey	MF-*Tilia* Honey	Goldenrod Honey	MF-Lamiaceae Honey
Brassicaceae	45.3	-	6.7	15.6
*Tilia*	3.2	21.5	-	1.6
*Solidago*	-	-	45.3	6.8
Lamiaceae	-	-	-	30.8
*Robinia*	5.3	12.1	2.7	1.6
Rosaceae	1.1	10.1	-	4.0
Asteraceae	-	10.7	20.4	5.6
Caryophyllaceae	-	4.7	-	-
Poaceae	-	1.3	4.9	-
Apiaceae	-	3.4	0.9	-
Liliaceae	5.3	-	-	-
*Fagopyrum*	-	-	-	3.6
*Trifolium*	-	-	-	1.2
Fabaceae	-	-	-	1.2
Others	40.0	29.5	24.9	23.2

Dominant pollen >45%, secondary pollen 16–45%, important minor pollen 3–15%, minor pollen <3% of the pollen grains counted.

**Table 3:** Total antioxidant capacities of the honey samples.

Nr.	Honey Types	TRC (mg GAE kg^−1^)	TEAC (µmol TE 100 g^−1^)	DPPH (IC_50_ mg mL^−1^)	ORAC (µmol TE g^−1^)
1	Milkweed	144.72 ± 17.17 ^a^	110.87 ± 3.80 ^a^	37.61 ± 0.41 ^a^	22.67 ± 0.97 ^a^
2	MF-*Tilia*	195.44 ± 9.87 ^ab^	124.35 ± 6.94 ^a^	37.16 ± 1.57 ^a^	63.00 ± 4.43 ^b^
3	Goldenrod	255.27 ± 22.44 ^b^	155.71 ± 7.91 ^b^	33.65 ± 2.20 ^ab^	19.50 ± 1.69 ^a^
4	MF-Lamiaceae	475.71 ± 40.63 ^c^	177.87 ± 4.20 ^b^	28.52 ± 0.81 ^b^	32.41 ± 2.41 ^c^

TRC—Total Reducing Capacity; TEAC—Trolox Equivalent Antioxidant Capacity; DPPH—Antiradical Power; ORAC—Oxygen Radical Absorbance Capacity; Data are means ± standard deviations of three independent determinations (*n* = 3). Data in the same column with different superscripted letters mean significant differences among various honeys according to Student’s *t*-test (*p* < 0.01).

**Table 4:** Macroelement content of the studied honey samples.

Nr.	Honey Types	K (mg kg^−1^)	Ca (mg kg^−1^)	P (mg kg^−1^)	S (mg kg^−1^)	Mg (mg kg^−1^)	Na (mg kg^−1^)
1.	Milkweed	340.28 ± 11.12 ^a^	19.06 ± 2.67 ^a^	39.19 ± 3.56 ^ab^	15.98 ± 1.88 ^ab^	11.78 ± 0.10 ^a^	6.38 ± 1.38 ^a^
2.	MF-*Tilia*	845.88 ± 35.67 ^b^	53.71 ± 11.05 ^b^	37.73 ± 2.69 ^a^	14.22 ± 0.94 ^a^	15.74 ± 1.30 ^b^	37.02 ± 8.49 ^b^
3.	Goldenrod	342.73 ± 12.29 ^a^	75.79 ± 10.44 ^c^	40.74 ± 3.85 ^b^	16.67 ± 1.15 ^b^	24.30 ± 0.11 ^c^	8.69 ± 0.36 ^c^
4.	MF-Lamiaceae	1264.73 ± 70.79 ^c^	73.78 ± 12.22 ^c^	127.04 ± 4.20 ^c^	52.31 ± 0.67 ^c^	34.54 ± 2.07 ^d^	8.80 ± 1.43 ^ac^

Data are means ± standard deviations of three independent measurements (*n* = 3). Data in the same column with different superscripted letters mean significant differences among various honeys according to Student’s *t*-test (*p* < 0.01).

**Table 5:** Microelement content of honey samples.

Nr.	Honey Types	B (mg kg^−1^)	Cu (mg kg^−1^)	Fe (mg kg^−1^)	Mn (mg kg^−1^)	Zn (mg kg^−1^)
1.	Milkweed	3.79 ± 0.63 ^a^	0.13 ± 0.01 ^a^	0.73 ± 0.00 ^a^	0.12 ± 0.03 ^a^	0.44 ± 0.00 ^a^
2.	MF-*Tilia*	2.41 ± 0.52 ^b^	0.13 ± 0.01 ^a^	0.62 ± 0.08 ^a^	0.62 ± 0.05 ^b^	0.63 ± 0.00 ^a^
3.	Goldenrod	4.90 ± 1.02 ^ac^	0.13 ± 0.01 ^a^	1.80 ± 0.60 ^b^	0.16 ± 0.01 ^a^	2.15 ± 0.20 ^b^
4.	MF-Lamiaceae	6.49 ± 0.43 ^c^	0.77 ± 0.03 ^b^	1.53 ± 0.29 ^b^	0.77 ± 0.01 ^c^	3.32 ± 0.04 ^c^

Data are means ± standard deviations of three independent measurements (*n* = 3). Data in the same column with different superscripted letters mean significant differences among various honeys according to Student’s *t*-test (*p* < 0.01).

**Table 6:** Correlation matrix (Pearson’s correlation coefficients) of color, antioxidant and multielement parameters in Hungarian honeys.

Variable	Color	TRC	TEAC	DPPH	ORAC
TRC	0.865 *				
TEAC	0.979 ***	0.912 **			
DPPH	0.894 ***	0.946 ***	0.948 ***		
ORAC	−0.322 ***	−0.087 ***	−0.237 ***	0.226	
K	0.477 *	0.774 ***	0.574 ***	0.628 ***	0.475 ***
Ca	0.842 ***	0.620 ***	0.778 ***	0.589 **	0.001 ***
P	0.716 ***	0.948 ***	0.786 ***	0.882 *	−0.078 ***
S	0.722 ***	0.943 ***	0.788 ***	0.880	−0.109 ***
Mg	0.961 ***	0.939 ***	0.967 ***	0.920 ***	−0.204 ***
Na	−0.362 ***	−2.275 ***	−0.329 ***	−0.417 ***	0.916 ***
B	0.794 ***	0.824 ***	0.815 ***	0.853 ***	−0.557 ***
Cu	0.674 ***	0.934 ***	0.755 ***	0.857 ***	0.011 ***
Fe	0.823 ***	0.652 ***	0.807 ***	0.708 ***	−0.418 ***
Mn	0.411 ***	0.689 ***	0.506 ***	0.523 ***	0.625 ***
Zn	0.964 ***	0.921 ***	0.973 ***	0.946 ***	−0.347 ***

TRC—Total Reducing Capacity; TEAC—Trolox Equivalent Antioxidant Capacity; DPPH—Antiradical Power; ORAC—Oxygen Radical Absorbance Capacity; significant at * *p* < 0.05, ** *p* < 0.01, *** *p* < 0.001.

## Data Availability

The data presented in this study are available on request from the corresponding author.

## Sample Availability

Honey samples are available from the authors.
